# A Machine-Learning Strategy to Detect Mura Defects in a Low-Contrast Image by Piecewise Gamma Correction

**DOI:** 10.3390/s24051484

**Published:** 2024-02-24

**Authors:** Zo-Han Lin, Qi-Yuan Lai, Hung-Yuan Li

**Affiliations:** Department of Mold and Die Engineering, National Kaohsiung University of Science and Technology, Kaohsiung 82445, Taiwan; 1104406101@nkust.edu.tw (Z.-H.L.); f110147135@nkust.edu.tw (Q.-Y.L.)

**Keywords:** machine-learning, display panel, Mura detection and classification, APSO, CNN

## Abstract

A detection and classification machine-learning model to inspect Thin Film Transistor Liquid Crystal Display (TFT-LCD) Mura is proposed in this study. To improve the capability of the machine-learning model to inspect panels’ low-contrast grayscale images, piecewise gamma correction and a Selective Search algorithm are applied to detect and optimize the feature regions based on the Semiconductor Equipment and Materials International Mura (SEMU) specifications. In this process, matching the segment proportions to gamma values of piecewise gamma is a task that involves derivative-free optimization which is trained by adaptive particle swarm optimization. The detection accuracy rate (DAR) is approximately 93.75%. An enhanced convolutional neural network model is then applied to classify the Mura type through using the Taguchi experimental design method that identifies the optimal combination of the convolution kernel and the maximum pooling kernel sizes. A remarkable defect classification accuracy rate (CAR) of approximately 96.67% is ultimately achieved. The entire defect detection and classification process can be completed in about 3 milliseconds.

## 1. Introduction

As display technology advances, TFT-LCD has emerged as the predominant technology in flat-panel display products. Its widespread adoption can be attributed to its inherent benefits, such as energy efficiency and slim profile. To enhance product quality and bolster profitability, panel manufacturers are unwavering in their commitment to reducing the production of defective units to reduce losses. The manufacturing procedures for TFT-LCD panels are multifaceted and intricate, featuring dedicated inspection equipment at each stage to scrutinize for any defects. These potential product defects fall into two categories: microscopic and macroscopic. Microscopic defects are typically smaller than 100 µm and cannot be visually detected by human personnel. These defects can only be captured through camera systems or magnification, followed by categorization by inspection personnel. Macroscopic defects are identifiable through visual inspection and often manifest as the most prevalent issues, such as uneven brightness and Mura, on the glass surface.

**Figure 1:**
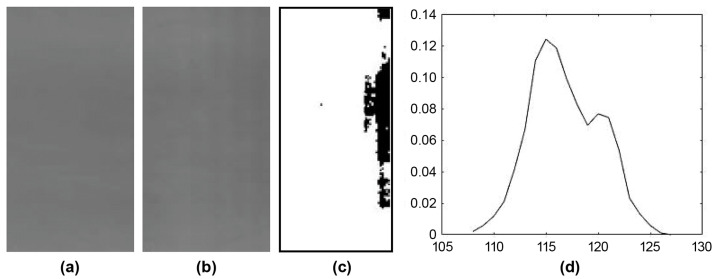
(**a**) An image of glass panel with no Mura; (**b**) An image of glass panel with Thin Mura (on the right); (**c**) Binarized image of (**b**); (**d**) Histogram of (**b**).

Mura defects can be categorized into three primary shapes: Dot, Thin, and Area types [1]. Figure 1a is a standard panel image devoid of Mura. Figure 1b illustrates an image of a panel with a Thin Mura defect on the right side of the panel. The grayscale value within the defect area registers around 110, while the background exhibits a grayscale value of approximately 125. This discrepancy is challenging to discern with unaided eyes. Figure 1c demonstrates the binarized version of the localized lateral Mura area from Figure 1b, revealing distinct black vertical lines. The histogram of Figure 1b is depicted in Figure 1d. In a grayscale range of 0 to 255, the digital grayscale image predominantly spans between 108 and 130. In contrast to the full 256 grayscale levels, this image confines itself to a mere 22 grayscale levels. Consequently, the image appears dark, characterized by low contrast and a narrow bandwidth.

Different approaches have been employed for quantitative Mura evaluation [2,3,4]. Chen et al. [5] applied filtering techniques to detect Thin-type Mura. Kim and Lee [6] detected Mura through the image histogram threshold based on Weber’s method. Furthermore, Chang et al. [7] employed Otsu binarization to identify the threshold that best distinguishes Mura from the background in a given environment. These study efforts encompass spatial and frequency domain image processing methods. The spatial domain method primarily determines the threshold distinction between Mura and its background, while the frequency domain approach is typically time-intensive. 

Several studies have attempted to use machine-learning methods for the automated detection of Mura. Mura defects on LCD panels are characterized by low contrast, overall dimness, and diversity and coexistence. Diversity and coexistence refer to the presence of various shapes of Mura defects that may coexist on the same LCD panel, thereby making it difficult for machine-learning to detect. Signh et al. compared the DAR of different deep-learning methods in 2019 [8]. The best result for the classification and localization of Mura defects using a state-of-the-art deep-learning network is F1~80%. Deep channel attention-based classification network (DCANet) was proposed by Lin et al. [9] as a feature extractor using an antagonistic training algorithm based on a convolution neural network. It was a data augmentation method for inconspicuous targets. Xie et al. [10] proposed a U-shape generator to detect Mura in a generative adversarial network (GAN), with a detection speed of ~5.6 ms. per frame and 256×256 resolution each. Moreover, the detection accuracy of defects with larger shapes was higher than that with smaller shapes. Other studies [11,12] have also used GAN to detect Mura on LCD frames. However, GAN is a binary classification model with no capability to classify different types of Mura. 

Machine-learning models other than GAN have been studied by many researchers. An automated Mura defect detection system using a random forest classifier was applied to classify different types of Mura defects, such as white and black background with line and region/shape Mura. The proposed system obtained the best accuracy results of > 99% but 27 ms. per image [13]. Furthermore, a convolutional neural network (CNN)-based transfer learning method was reported by Imoto et al. with a defect detection accuracy of ~90% on a semiconductor device observed using SEM [14]. Other neural network (NN) models have also been reported, including back-propagation NN [15] and RetinaNet [16]. However, most of the region-based convolutional neural network (R-CNN) models developed require human labor to select regions, which is difficult in its nature owing to the low contrast, diversity, and complexity of Mura defects on LCD frames.

The spatial enhancement Mura unification (SEMU) specification proposed by the International Semiconductor Equipment and Materials International (SEMI) in 2002 (SEMI Mura, SEMI D31-1102) [17] serves as a fundamental tool for assessing the extent of Mura on flat-panel displays. 

To enhance low-contrast grayscale images, piecewise linear function transformation and nonlinear function transformation techniques [18,19,20,21,22,23] are often used. Nonlinear function conversion uses a nonlinear operator applied to the normalized grayscale values of an image. In 2021, Pattanayak et al. [21] highlighted that a single gamma-based nonlinear conversion method might limit the grayscale improvement range in dark areas while causing the grayscale values in bright areas to increase excessively, resulting in poor contrast between the dark and bright regions in the image. Hence, a bi-linear piecewise mapping function was introduced to address this issue of grayscale contrast within specific areas, with a fixed normalized grayscale value serving as the boundary. Yan et al. [23] also proposed enhancing the grayscale contrast of Mura using gamma correction.

However, images featuring Mura chromatic aberration defects pose challenges, as their grayscale values are not constant. Consequently, this study introduces piecewise gamma correction (PGC) to adapt to an optimization algorithm for partition processing and weight evaluation. This presents a derivative-free optimization challenge, and so Adaptive Particle Swarm Optimization (APSO) is employed for solving such problems. Particle Swarm Optimization (PSO), initially proposed by Kennedy and Eberhart in 1995 [24], was subsequently enhanced by Shi and Eberhart in 1998 [25]. Zhan et al. [26] proposed APSO in 2009 by introducing enhancements to the standard PSO for attaining global optimal solutions easily. Tong et al. [27] introduced an exponential inertia weight reduction strategy based on the distance between particles, which expedited the iteration speed.

To identify a specific object within an image, an initial search of all conceivable candidate regions is imperative. Subsequently, these candidate regions are assessed to be those that exhibit the desired target characteristics. Selective search (SS) was introduced by Uijlings et al. in 2013 [28]. SS significantly diminishes the requisite number of searches while concurrently reducing the computational time. Based upon this foundation, many neural networks based on R-CNN, such as Fast R-CNN [29], Faster R-CNN [30], and You Only Look Once (YOLO) [31], continue to employ the SS method, solidifying its relevance in this domain.

The regional neural network consists of two components: one to search for and the other for the classification of candidate areas. The CNN model, as one of the most important classification methods, was initially introduced by Yann LeCun et al. in 1998 [32]. Over time, it was refined for the development of deep-learning architectures [33]. Weimer et al. [34] proposed a classification approach for six distinct panel defects and fine-tuned the CNN hyperparameters, such as the quantity of kernels in each layer and the number of filters in each layer within the CNN model, to enhance classification accuracy.

Owing to Mura’s low-contrast characteristics, its identification is challenging, and a single panel can exhibit multiple intricate and difficult-to-extract defects simultaneously.

This study proposes a computational model integrating detection and classification to enhance Mura defect detection capabilities. In the detection phase, panel image contrast is first enhanced using PGC with a flexible gamma value calculated by the black-box optimization algorithm, APSO. SS is introduced to select all possible regions by color similarity, and an SEMU threshold determined statistically is employed to filter out false regions. A pre-classified image database is exercised, and the DAR is used as the objective function in the APSO. These detection phases evolved until reaching the maximum number of iterations or finding the maximum DAR.

CNN is then applied to classify those identified defect regions and the hyperparameters of the CNN model are fine-tuned using Taguchi experimental design techniques to enhance the CAR.

## 2. Research Theories and Methods

Section 2 presents the research background of SEMU, PGC, APSO, and CNN. Additionally, the relevant parameters and study structure are detailed.

### 2.1. Semiconductor Equipment and Materials International Mura Formula

SEMU is a standard defined by SEMI to evaluate the Mura index, as shown in Equation (1) [17].

(1) $$SEMU=\frac{\left|C_{x}\right|}{C_{jnd}}=\frac{\frac{\left|I_{m}-I_{b}\right|}{I_{b}}}{\left(\frac{1.97}{A^{0.33}}+0.72\right)},$$

$C_{x}$: The ratio of gray-level differences between the Mura area and the surrounding non-Mura region.

$C_{jnd}$: Grayscale contrast value of Mura.

$I_{m}$: The average grayscale value within the Mura region.

$I_{b}$: The average grayscale value of the non-Mura area (background).

A: The area of the Mura region (mm).

In Equation (1), the greater the grayscale difference ratio $C_{x}$, the more pronounced the grayscale disparity of the Mura defect and the easier it is to discern. Additionally, a larger defect area A enhances the likelihood of detection. In this study, the input image was first segmented using the segmentation method [35] and then the hierarchical grouping algorithm was employed to merge the initially segmented neighboring region pairs, primarily relying on color similarity, which can result in a region proposal. The color similarity strategy initially divides the image’s grayscale levels into 25 bins in the histogram, and the color similarity between any neighboring region pair is calculated as shown in Equation (2) [28]:

(2) $$SS_{colour}\left(r_{\alpha },r_{\beta }\right)=\sum_{k=1}^{n}\min\left(c_{\alpha }^{k},c_{\beta }^{k}\right),$$

$r_{\alpha }:Region\;\alpha .$

$r_{\alpha },r_{\beta }:$ Neighboring region pair.

$c_{\alpha }^{k}:\;$The k-th bin value of region *α* in the color histogram. 

Equation (2) calculates the values of color histograms within neighboring region pairs, denoted as $r_{\alpha }and{\;r}_{\beta }$, then merges the most similar region pairs in the segmented regions. This process continues iteratively until no neighboring regions remain, ultimately resulting in the formation of a regional proposal. 

### 2.2. Piecewise Gamma Correction

Conventional Gamma Correction (CGC) is commonly applied to the normalized grayscale values of an image to boost grayscale contrast while preserving the grayscales of the brightest and darkest areas. However, it is less effective for enhancing the contrast in low-contrast images. This study introduces a method for enhancing the contrast between bright and dark regions in images. PGC is an extension of CGC.

In this study, PGC is employed to partition the grayscale values within the image into specific segments, and different gamma values are used to enhance the contrast of each segment. PGC is defined as shown in Equation (3).

(3) $$S_{j}=C_{j}R^{\gamma_{j}},$$

S: Output grayscale normalized value.

$C_{j}$: Constant for each segment.

R: Input grayscale normalization value.

$\gamma_{j}$: Gamma for each segment.

j: Segment index.

**Figure 2:**
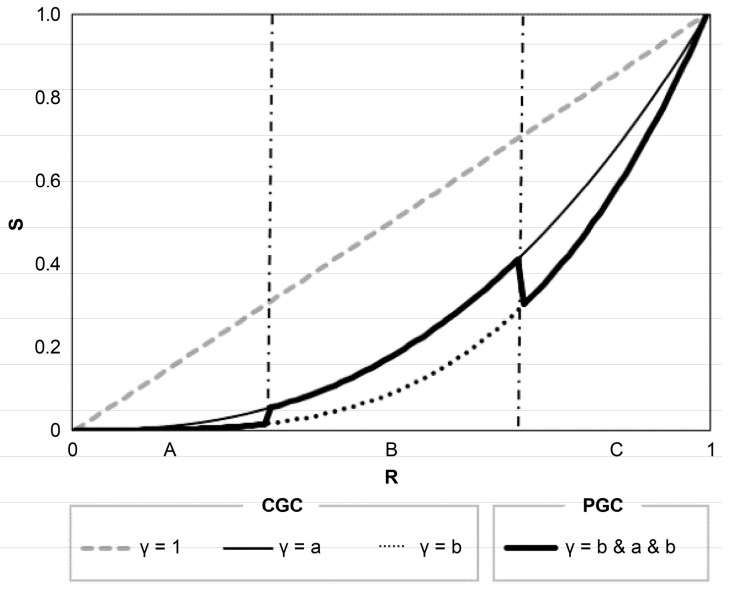
CGC and PGC comparison between input and output. Segments A, B and C on the horizontal axis represent different grayscale value areas on the histogram.

Figure 2 presents a comparison between CGC and PGC. The thick lines represent the enhanced output of PGC. As illustrated in Figure 2, PGC has the ability to independently boost the grayscale contrast within specific segments, offering greater flexibility compared to CGC. Through the use of a proper optimization algorithm, the size of each segment and its associated gamma value can be calculated.

### 2.3. Adaptive Particle Swarm Optimization

PSO was originally proposed using the example of a flock of foraging birds, with each bird likened to a particle [14]. These birds determine the speed and direction of their next flight based on their current state. At each iteration, the group’s position is considered as a potential solution. If the position has reached the best solution, the group stops moving; otherwise, they continue searching for the next move. Each particle has its “local best” (pbest), and the best position found by all particles in the group is termed the “global best” (gbest). The particle update rate and position follow Equations (4) and (5) [15].

(4) $$v_{i}^{t+1}=w\times v_{i}^{t}+c_{1}{rand}_{1}\left(p_{i}^{t}-x_{i}^{t}\right)+c_{2}{rand}_{2}\left(p_{g}^{t}-x_{i}^{t}\right),$$

(5) $$x_{i}^{t+1}=x_{i}^{t}+v_{i}^{t+1},$$

w: Inertia weight value.

$x_{i}^{t}$: The position of particle *i* at time *t*. 

$v_{i}^{t}$: The flight velocity vector of particle *i* at time *t*.

$p_{i}^{t}$: The local best position pbest searched by particle *i* at time *t*.

$p_{g}^{t}$: The global best position gbest searched by the particle swarm at time *t*.

${rand}_{1}$: Random value, $\in \left[0,1\right]$.

${rand}_{2}$: Random value, $\in \left[0,1\right]$.

$c_{1}$: Individual learning factor.

$c_{2}$: Group learning factor.

Equation (4) outlines how a particle updates its speed by considering the vector distance between the speed from the previous iteration, its current position, its individual best position, and the best position of the entire group. Equation (5) describes how the particle relocates to a new position. In these equations, the position vector of particle *i* in dimension *d* at time *t* is represented as $x_{i}^{t}=\left(x_{i1}^{t},x_{i2}^{t},\ldots ,x_{id}^{t}\right)$, where $x_{id}^{t}\in \left[L_{d},U_{d}\right]$, and $L_{d}$, and $U_{d}U_{d}$ signify the lower and upper bounds in the search space, acting as boundary constraints.

PSO requires users to define specific parameter values, and these parameters directly impact the rate of convergence and the potential for falling into local optimal solutions. In 2009, Zhan et al. [16] introduced APSO, which employs three techniques: evolutionary state estimation, adaptive control of PSO parameters, and elite learning strategy (ELS), to enhance PSO’s convergence speed and accuracy.

Among these strategies, ELS introduces a solution through a search increment based on Gaussian perturbation. However, there is a risk that perturbed parameters may extend beyond the designated search space. The typical remedy is to confine the parameters within the upper and lower bounds directly, but this often causes particles to reach these boundaries prematurely, leading to monotonic parameter combinations. In this study, we propose Gaussian perturbation with proportional characteristics, as described in Equation (6), as a proportional factor to prevent parameter values from exceeding the search space boundaries. The scaling factors are outlined in Equations (7) and (8).

(6) $$p_{i}^{d}=p_{i-1}^{d}+\left(x_{max}^{d}-x_{min}^{d}\right)\times Gaussian\left(\mu ,\;\sigma^{2}\right)\times sf,$$

(7) $$sf=\frac{x_{max}^{d}-p^{d}}{\left(x_{max}^{d}-x_{min}^{d}\right)\times 2},$$

(8) $$sf=\frac{p^{d}-x_{min}^{d}}{\left(x_{max}^{d}-x_{min}^{d}\right)\times 2},$$

sf: Proportional factor.

$p_{i}^{d}$: Correction variable in dimension D.

$x_{max}^{d}$: Upper limit on search space.

$x_{min}^{d}$: Lower limit on search space.

### 2.4. Convolution Neural Network

The CNN basic model architecture consists of two core blocks: feature extraction and classification. Feature extraction is responsible for extracting image features through a sequence of operations, including the convolution layer, pooling layer, and flattening. The convolution layer employs various kernels to extract image features, resulting in feature maps. These features then undergo pooling to reduce their size, and then the output is flattened into one-dimensional data. In the classification stage, the one-dimensional data are processed using activation functions, optimized weights, and biases in the hidden layer of a multi-layer perceptron (MLP) and are finally classified.

### 2.5. Study Structure

**Figure 3:**
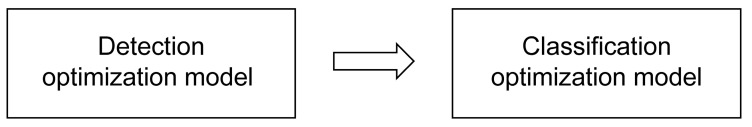
Study structure. The structure comprises two stages: DOM and COM.

The study structure is divided into two parts: the Detection Optimization Model (DOM) and the Classification Optimization Model (COM), as depicted in Figure 3. The purpose of the DOM is to optimize the positions of each segment in the PGC preprocessing and the gamma values utilized in each segment. This optimization method employs APSO to ultimately achieve the highest DAR. The purpose of the COM stage is to classify different Mura types. The Taguchi experimental design method is used to optimize the specific hyperparameters of the CNN model and to ultimately achieve the highest CAR. 

**Figure 4:**
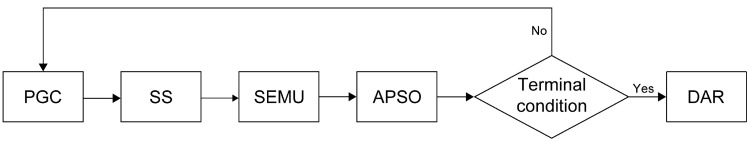
DOM flow chart.

In the DOM, as shown in the flowchart provided in Figure 4, the process begins with inputting the pre-classified image, where PGC and a 5 × 5 mean smoothing blur filter are applied for image processing. Due to the low grayscale difference of Mura images, PGC is employed to selectively increase the contrast of specific grayscale values. The image histogram is divided into three segments; each is enhanced with a different gamma value. After PGC, noise may appear, and a blur filter is applied to reduce noise and avoid the impact on the detected regions by SS. Subsequently, SS is utilized to search for all possible Mura regions by a color similarity strategy. All regions are then scored and filtered by the SEMU threshold. The threshold of SEMU indicates the Mura inspection specifications, with a smaller threshold indicating stricter inspection criteria. The partition size of the PGC and the gamma value for each region are configured as design variables for optimization. The objective is to find the highest DAR using APSO. Before reaching the termination condition, APSO will repeatedly return to the PGC step until reaching the maximum number of iterations or finding the maximum DAR, at which point the process stops.

Grayscale images utilized in this study are sourced from a particular panel company. Prior to the study, dedicated panel experts categorized the images into four distinct types based on Mura characteristics, namely No Mura, Dot Mura, Thin Mura, and Area Mura. Each type comprises 100 images, totaling 400, with an image size of 200 × 382 pixels each.

In the COM stage, CNN is used as a classifier. As described in Section 2.4, the CNN model in this experiment includes three integrated layers, each comprising a convolutional layer and a maximum pooling layer. The main objective of the COM stage is to optimize the hyperparameter values selected in the CNN model to improve the CAR. During the optimization process of the CNN model, specific hyperparameters, such as the kernel size of the convolutional layer and the maximum pooling layer, are treated as experimental factors within the fixed CNN training architecture. A total of 6 kernel sizes were selected from the convolutional layers and the maximum pooling layers, with two levels in each kernel. Conducting a full factorial experiment would require experiments with 64 combinations. Therefore, Taguchi’s experimental design method was employed to conduct 12 experimental combinations. From the experimental results of these 12 combinations, the combination that achieves the maximum CAR among all combinations is determined. The Mura regions identified by the DOM are fed into the COM which uses the CNN model for training.

### 2.6. Mura Region Type Classification

Table 1 outlines the classification criteria for Mura regions based on size. Regions identified by SS are categorized as different Mura types based on these criteria. The DAR is determined by comparing the detected and classified regions with the conditions outlined in the original tested image category, using 100 images for each of the four types for verification.

### 2.7. Piecewise Gamma Correction—Segmented Regions and Boundary Conditions

**Figure 5:**
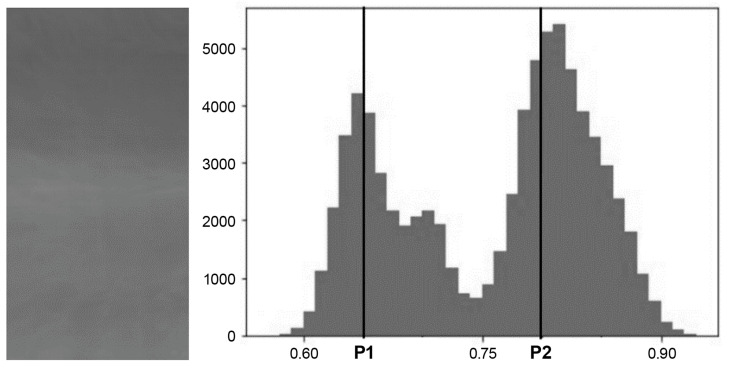
Mura original image and its normalized histogram.

To establish the segmented size and boundary conditions for PGC, it is imperative to identify the specific features of the image to be highlighted. This process involves transforming the original image with Mura into a normalized histogram, as illustrated in Figure 5. The grayscale value within the image exhibits a bimodal distribution. To effectively employ PGC to enhance the two peaks and the lower segments between them using varying gamma values, the brightness is divided into three distinct segments based on two points, P1 and P2. The values of P1 and P2 adhere to the condition 0 < P1 < P2 < 1. Through the optimization algorithm, the optimal positions of P1 and P2, along with the gamma values for each of these three segments, can be determined.

### 2.8. APSO Set Parameters and Terminal Conditions

Table 2 outlines the parameter settings and terminal conditions for APSO. The number of variables encompasses the gamma values for the three segments in PGC and the segmentation points P1 and P2. The optimization process is subject to three terminal conditions, with the process terminating if any of these conditions are met.

### 2.9. CNN Training Materials

**Figure 6:**
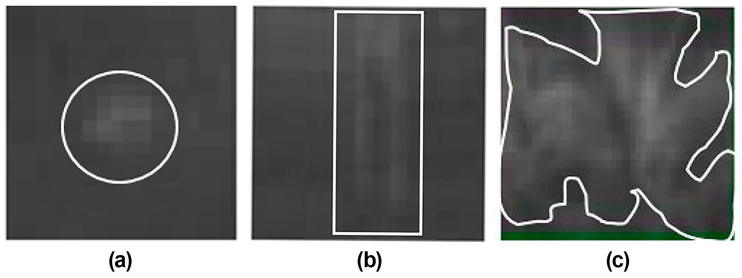
(**a**) Dot Mura; (**b**) Thin Mura; (**c**) Area Mura. The defects in the figures were image-processed. The white lines represent the boundary of Mura.

The region images employed in the CNN training model consist of the three Mura types (Dot, Thin, and Area) selected from the DOM optimization model. The resolution of the region image was 200 × 200 (pixels) after resizing. Among the Mura region samples, 1300, 4500, and 4500 Mura images were obtained and classified as Dot, Thin, and Area Mura, respectively. Figure 6 shows three types of images: (a) image with Dot Mura, (b) image with Thin Mura, and (c) image with Area Mura. 

### 2.10. CNN Training Model Description

**Figure 7:**
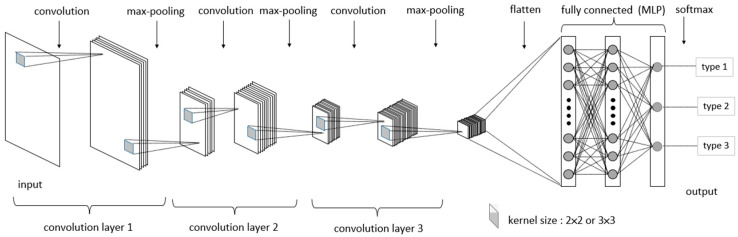
CNN model architecture.

The CNN model architecture used in this study, as depicted in Figure 7, incorporates three integrated layers, each comprising a convolutional layer and a maximum pooling layer within the image feature extraction section. The extracted features are flattened and connected to an MLP. In the MLP, rectified linear unit excitation functions are employed, and final classification is performed using the softmax function.

### 2.11. CNN Hyperparameter Settings

The configuration of hyperparameters in the training model significantly impacts the model’s accuracy. This study primarily focuses on the convolution kernel size and Max-Pooling kernel size as the hyperparameters of interest. The optimization of CNN hyperparameters is carried out in conjunction with Taguchi’s experimental method, where six factors, labeled as A-F, detailed in Table 3, are selected. Each factor has two levels. The highest CAR is the expected result. CAR is defined as the classification accuracy of 100 images which are not in the training dataset, each of 200 × 382 pixels, for Dot, Thin, and Area Mura. These images are classified using the trained CNN model to assess the classification accuracy for these 300 images. The other fixed hyperparameter settings are presented in Table 4.

During the preliminary testing phase, it was observed that when convolution kernel and Max-Pooling kernel sizes are set to 4 × 4, the resulting image becomes too small, rendering the program unable to generate the required feature map. The convolution and Max-Pooling kernel sizes are therefore set to 2 × 2 and 3 × 3, respectively. 

Due to limitations in image resolution in this experiment, using only two layers resulted in a CAR of less than 70%, while using four layers caused the CNN training process to terminate due to the resulting image being too small for training. Therefore, experiments were conducted using only three layers. This three-layer architecture was also found in the study by Weimer et al. [34]. 

Each combination is subjected to three repetitions to mitigate the impact of uncontrollable factors on the experimental results. Ultimately, based on these experimental results, the best and the worst-performing combinations are identified and compared. 

In this study, the experimental models of PGC, APSO, and CNN were developed in-house utilizing Python as the development environment, with Jupyter Notebook as the editor. The hardware specifications include a WIN10 computer equipped with an i5 1.8 GHz CPU, 12 GB RAM, and a GeForce MX150 NVIDIA GPU.

## 3. Experimental Results and Discussion

Section 3 presents the following four experimental results: 1. Determination of SEMU threshold. 2. Comparison of the effectiveness of PGC. 3. APSO experimental results of optimized training mode. 4. Experimental results of the CNN training model.

### 3.1. Determination of SEMU Threshold

**Figure 8:**
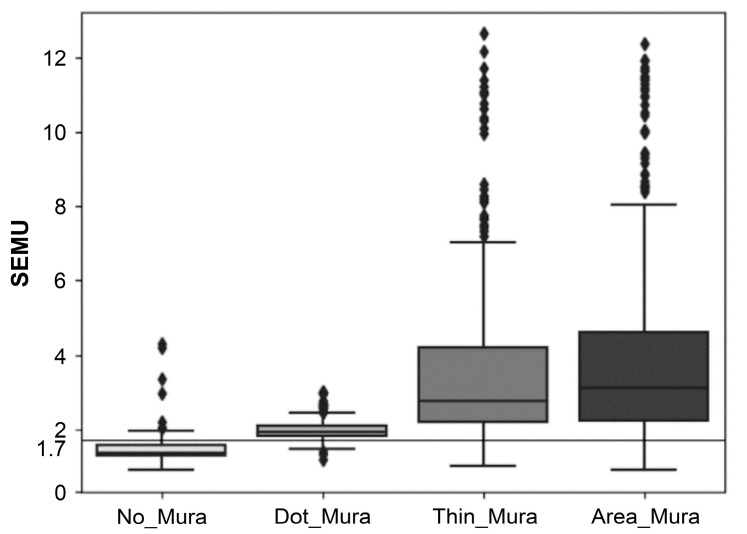
SEMU numerical distribution of four types of Mura regions.

Mura’s SEMU specifications at the panel factory are subject to changes in customer product standards. It is important to note that the SEMU threshold is not a fixed absolute value. A breakdown of the various region types and their SEMU value distributions is shown in Table 5. Figure 8 illustrates the SEMU value distribution of the four types of Mura regions identified in Table 5 using a box plot. The y-axis corresponds to the SEMU value, while the x-axis displays the regions from left to right: No Mura, Dot Mura, Thin Mura, and Area Mura. 

In the box plot, the five horizontal lines within each of the groups represent, from bottom to top, the minimum value, the first quartile (25%), the median (50%), the third quartile (75%), and the maximum value of the SEMU. Points below the minimum value and above the maximum value are considered outliers. Figure 8 shows that the distributions of regions in the first group (No Mura) and the second group (Dot Mura) are relatively close. Therefore, when selecting regions from the first group (No Mura), avoiding excessive deletion of Dot Mura regions is essential, as this could result in excluding images containing actual regions of Dot Mura. Regions with SEMU values below the SEMU threshold will be removed, and so the selection of the SEMU threshold must align with the proportion of Mura regions deleted by varying SEMU values.

Table 6 presents the proportion of deleted Mura regions with varying SEMU values. As observed in Table 6, selecting an SEMU threshold of 1.7 results in deleting around 90% of the pseudo-Mura regions in Group A (No Mura) while deleting less than 10% of actual Mura regions from other groups. Therefore, setting the SEMU threshold at approximately 1.7 aligns with the intention to retain more genuine Mura regions from categories B–D while eliminating more false Mura regions to improve DAR.

### 3.2. Comparison of the Effectiveness of PGC

**Figure 9:**
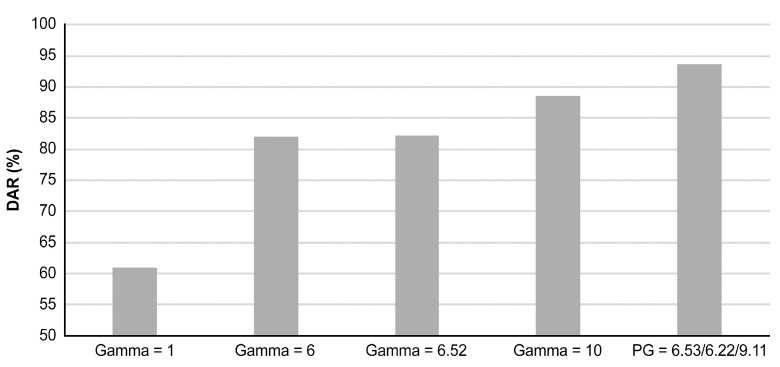
DAR comparison of PGC and CGC.

To explore the effectiveness of PGC, a comparison was made between the PGC and CGC. Figure 9 illustrates the DAR obtained by inputting gamma values into the Mura detection model on the y-axis, while the x-axis represents five distinct gamma groups. The first group on the left corresponds to gamma = 1, and represents no gamma correction. The second to fourth groups indicate single gamma values, specifically 6, 6.52, and 10. The values 6 and 10 also represent gamma’s lower and upper limits within the optimization process. The value 6.52 results from fitting the PGC using the nonlinear least squares method, yielding a coefficient of determination $R^{2}$ of 0.984. The fifth group employs PGC, with the values representing experimental results from APSO: 6.53, 6.22, and 9.11, respectively. The figure demonstrates that the PGC achieves the highest DAR, while the lowest DAR is observed when no power law is applied. This validates a significant enhancement in the Mura detection accuracy of PGC compared to CGC. 

### 3.3. APSO Experimental Results of Optimized Training Model

APSO is employed to optimize the detection model as finding each segment size and their associated gamma values in PGC represent an issue of derivative-free optimization. Table 7 provides the results of experiments conducted over 10 iterations of APSO optimization. This study involves three regions and two division points, denoted as P1 and P2. Within the table, G1, G2, and G3 correspond to the gamma values utilized in these three regions, namely 0 to P1, P1 to P2, and P2 to 1. Validation rate involves a comparison with the original 400 images to determine if the detected regions adhere to the conditions of the original image categories. DAR is evaluated using four different sets made up of 100 images each, all distinct from the training dataset, to assess the accuracy of the training model. Notably, these 400 images used for testing are categorized in the same manner as those employed in training.

The parameters within Group 7 achieve the highest validation rate of 96.75%, as indicated in Table 6. In this configuration, the gamma values for G1, G2, and G3 are 6.53, 6.22, and 9.11, respectively, with the segmentation points P1 and P2 positioned at 0.39 and 0.84. These results reveal that the gamma values in the first and second segments are similar. In contrast, the gamma values in the third segment exhibit a relatively drastic change. This also suggests that the Mura defects in this study’s 400 panel training samples tend to be biased toward brighter colors. Consequently, assigning a larger gamma value to the third segment effectively enhances the color contrast.

Using this trained Mura detection model, the DAR of 93.75% is achieved, with a total of 25 errors in region classification. A classification breakdown is presented in Table 8, which includes 14 errors in Class A (No Mura), in line with the SEMU threshold of 1.7, designed to preserve approximately 10% of regions related to pseudo-Mura. The remaining three types of authentic Mura all surpass a DAR of 94%, underscoring the model’s ability to effectively detect the majority of Mura defects.

**Figure 10:**
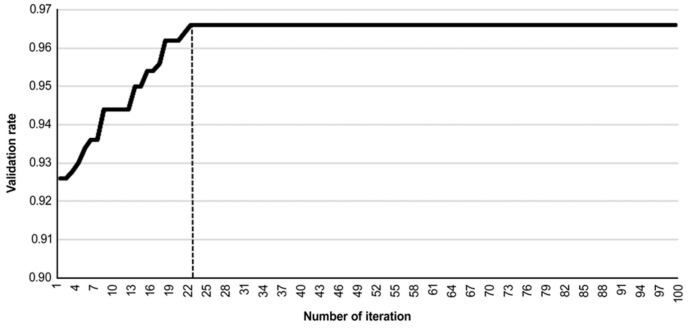
APSO training process validation rate and number of iterations. Dashed line in the figure represents convergence state is reached.

Figure 10 illustrates the convergence status of the validation rate and the number of iterations conducted during the training process. The training process converges at the 22nd iteration.

**Figure 11:**
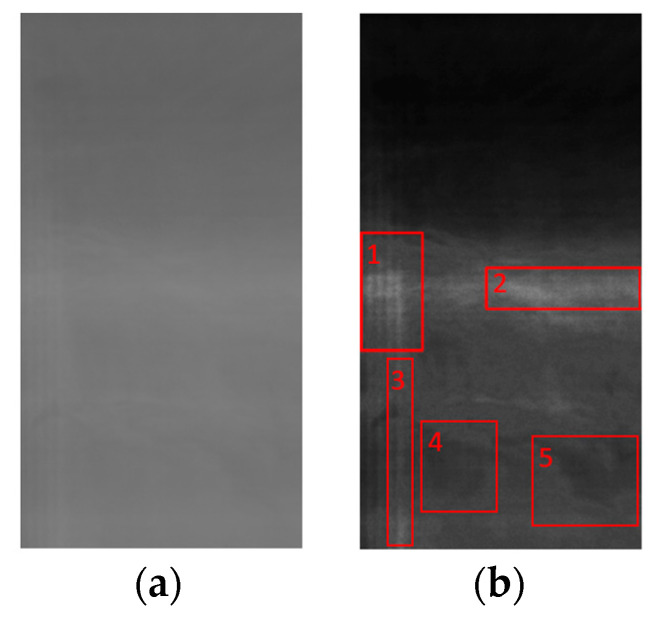
Mura images detected before and after. The image (**a**) represents the original image and image (**b**) shows the result of the original image after processing by the Mura detection model. Each detected Mura region is numbered in numerical order.

The optimal Mura detection model was obtained after optimizing the PGC segment positions and gamma values. Figure 11 displays the original image of Mura defect panels and the resulting image after processing by the Mura detection model. The red rectangles indicate the regions detected by the Mura detection model. Figure 11a includes both Thin and Area types of Mura. Figure 11b is the resulting image after processing by the detection model from the original Figure 11a. In Figure 11b, regions 1, 2, 4, and 5 belong to the Area-type Mura, while region 3 is the Thin-type Mura. Although region 1 exhibits both Thin and Area types, it can be classified as the Area type based on the Mura region classification conditions. From the original images, it can be observed that Mura defects have characteristics, such as low contrast, overall dimness, and diversity and coexistence. The results of the DOM phase in this study can overcome these Mura characteristics.

### 3.4. Experimental Results of the CNN Training Model

The CNN experimental procedure involves three runs, and then the average is taken. Table 9 presents the 12-factor combinations used according to the L^12^ orthogonal table and the CARs and their average values for the three repeated experiments.

### 3.5. Evaluation of the Effect of Main Factors

**Figure 12:**
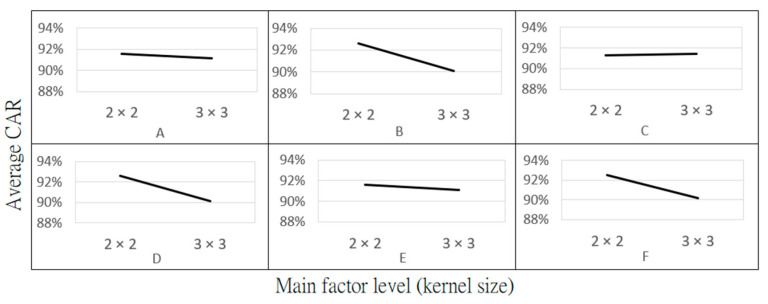
Main factor-level response to the average CAR. Main factor level refers to the two levels of each experimental factor from A to F.

The experimental results from Table 9 were subject to a main factor effect analysis, as shown in Figure 12, which illustrates the relationship between each factor level and the average CAR. The steeper the slope of each main effect, the greater the influence of that factor level on the model’s average CAR. The main effect values for each factor can be found in Table 10.

Based on Figure 12 and Table 11, factor B (Max-Pooling layer kernel size) in the first layer significantly impacts the average CAR. These results can also be explained by observing the changes in image size after each layer, as shown in Table 11. When the original input image passes through a convolution layer with a kernel size of 2 × 2 in the first layer, the image size reduces from 200 × 200 to 199 × 199 pixels. Subsequently, after passing through a Max-Pooling layer with a kernel size of 2 × 2, the image size decreases from 199 × 199 to 99 × 99 pixels. If both the convolution and Max-Pooling layers in the first layer use a 3 × 3 kernel size, the image size would be reduced to only 66 × 66. The substantial changes in image size highlight the significant impact of the first layer’s Max-Pooling kernel size on the average testing rate. The results also indicate that compared to using a 3 × 3 kernel size, a 2 × 2 kernel size in the Max-Pooling of the first layer preserves more detailed Mura feature characteristics.

### 3.6. Recommended Combination and Contrast Combination

Based on the main factor effect analysis results in Figure 12, the recommended combination (A-1, B-1, C-2, D-1, E-1, and F-1) can be used to achieve a better CAR. A contrasting combination (A-2, B-2, C-1, D-2, E-2, and F-2) is used for comparison, as shown in Table 12. The recommended and contrasting combinations are subjected to five repeated tests. The resulting CARs and their averages are presented in Table 13. The average test result for the recommended combination is 95.13%, representing an improvement of approximately 6.4% compared to the contrasting combination’s 88.73%. The highest CAR value is about 96.67%. The detection of Mura types in each 200 × 382 pixel image takes approximately 3 milliseconds.

## 4. Conclusions

This study focuses on detecting and classifying different types of Mura defects in the TFT-LCD manufacturing process. In the detection model, the SEMU scoring guideline is used to adjust the Mura inspection criteria, with the reference SEMU threshold of 1.7 used in this study. Furthermore, this study proposes a PGC segmentation method to enhance the grayscale contrast in the images. The optimal segmentation proportions and associated gamma values are obtained through the APSO algorithm. The highest DAR of ~93.75% using a PGC model is achieved, surpassing that of around 82.25% obtained using a CGC model. The results indicate the advantage of PGC in enhancing images with low contrast and narrow bandwidth.

For the classification model, the Taguchi experimental method was employed to identify the best combination of hyperparameters that enhance its classification capability. Experimental results show that the three most important factors affecting the CNN are all related to the kernel size in the maximum pooling layers, with importance decreasing from the first to the third layer. The proposed optimal CNN classification model achieves an average CAR of approximately 95.13%.

Combining the APSO-optimized PGC for the Mura defect detection model and the optimized CNN model for classification effectively enhances the detection and CAR of panel defects, with narrow bandwidth and low-contrast characteristics. This study can achieve a CAR of about 96.67% within 3 milliseconds.

In future research, consideration could be given to incorporating more hyper-parameters as experimental conditions to investigate whether a higher CAR can be achieved.

## Figures and Tables

**Table 1:** Mura Region Classification Conditions.

Mura Types	Mura Region Classification Logic
Dot Mura	The length and width of Mura region are ≥2 mm and ≤15 mm, respectively, with an aspect ratio ≤ 1.5.
Thin Mura	The Mura region width is between ≥2 mm and ≤15 mm, with no limit on length, with an aspect ratio ≥ 1.5.
Area Mura	The length and width of Mura region are both >15 mm, with an unlimited aspect ratio.

**Table 2:** APSO Set Parameters and Terminal Conditions.

APSO Algorithm Element	Setting
Objective function	Detection accuracy rate
Variable	P1, P2, G1, G2, G3
Constraint: P1, P2	0 < P1 < P2 < 1
Constraint: G1, G2, G3	∈[6, 10]
Initial Number of particles	50
Maximum number of iterations	100
Initial inertia weight w	0.9
Inertia weight w range	∈[0.4, 0.9]
Individual learning factor $c_{1}$	2.0
Group learning factor $c_{2}$	2.0
Experience weight $c,c_{1},c_{2}$ range	$c_{1},c_{2}$ ∈ [1.5, 2.5], $c_{1}+c_{2}$ ∈ [3, 4]
Terminal Conditions	
The number of iterations reaches the maximum: 100.	
The variation in the optimal solution position is less than 1 × 10^−8^.	
The optimal solution fitness value change is less than 1 × 10^−8^.	

**Table 3:** Experimental Design Factors.

Each Factor Has Two Levels	Convolution Kernel Size	Max-Pooling Kernel Size
Layer 1	A	B
Layer 2	C	D
Layer 3	E	F

**Table 4:** Fixed Hyperparameter Settings.

Fixed Hyperparameter	Setting
Number of layers	3
Dropout	30%
Dense	512
Activation function	ReLU
Epoch	10
Batch Size	10
Validation split	15%

**Table 5:** Number of Various Types of Regions and Distribution of SEMU Values.

Image Type	Region Number	SEMU Avg.	Min.	Max.	Median
A: No Mura	171	1.46	0.95	4.29	1.37
B: Dot Mura	216	2.01	1.31	3.02	1.92
C: Thin Mura	489	3.49	1.02	12.64	2.77
D: Area Mura	456	3.89	1.03	12.37	3.13

**Table 6:** Proportion of Deleted Mura Regions with Different SEMU Values.

	Proportion of Deleted Mura Regions in Each Category (%)			
SEMU	A: No Mura	B: Dot Mura	C: Thin Mura	D: Area Mura
1.37	50%	0.46%	3.27%	1.54%
1.47	60%	1.39%	4.91%	3.07%
1.52	70%	1.85%	4.91%	4.82%
1.59	80%	5.09%	7.16%	5.92%
1.7	90%	9.26%	10.02%	7.46%
4.29	100%	100%	76.07%	71.05%

**Table 7:** APSO Optimization Model Experimental Results.

Combination	Gamma Value			Segmentation Point		Validation Rate	DAR
	G1	G2	G3	P1	P2		
1	7.57	9.5	9.98	0.73	0.87	96.25%	92.50%
2	6.19	7.84	7.99	0.72	0.86	96.25%	92.25%
3	6.83	6.02	9.76	0.01	0.68	95.75%	91.00%
4	8.63	7.97	9.47	0.36	0.68	95.75%	91.50%
5	6.42	6	9.18	0.29	0.68	96.50%	93.25%
6	6.81	6.6	9.96	0.24	0.66	95.50%	91.25%
7	6.53	6.22	9.11	0.39	0.84	96.75%	93.75%
8	6.84	6.51	9.66	0.26	0.69	96.00%	93.25%
9	6.82	6	8.69	0.31	0.67	94.50%	90.75%
10	8.63	7.97	9.46	0.37	0.68	95.25%	91.75%

**Table 8:** DAR of Each Category of Mura Region.

Image Type	Number of Images	Correct Quantity	Incorrect Quantity	DAR
A: No Mura	100	86	14	86%
B: Dot Mura	100	94	6	94%
C: Thin Mura	100	98	2	98%
D: Area Mura	100	97	3	97%
Average DAR				93.75%

**Table 9:** Three L^12^ Experimental Results of CAR.

Combination	A	B	C	D	E	F	Experimental Results (CAR)			Average
1	2 × 2	2 × 2	2 × 2	2 × 2	2 × 2	2 × 2	96.67%	95.00%	96.33%	96.00%
2	2 × 2	2 × 2	2 × 2	2 × 2	2 × 2	3 × 3	94.00%	94.67%	89.67%	92.78%
3	2 × 2	2 × 2	3 × 3	3 × 3	3 × 3	2 × 2	95.33%	96.33%	80.00%	90.55%
4	2 × 2	3 × 3	2 × 2	3 × 3	3 × 3	2 × 2	89.67%	92.00%	90.00%	90.56%
5	2 × 2	3 × 3	3 × 3	2 × 2	3 × 3	3 × 3	92.33%	87.67%	93.33%	91.11%
6	2 × 2	3 × 3	3 × 3	3 × 3	2 × 2	3 × 3	76.33%	93.67%	94.67%	88.22%
7	3 × 3	2 × 2	3 × 3	3 × 3	2 × 2	2 × 2	90.00%	96.67%	93.67%	93.45%
8	3 × 3	2 × 2	3 × 3	2 × 2	3 × 3	3 × 3	91.67%	91.33%	93.67%	92.22%
9	3 × 3	2 × 2	2 × 2	3 × 3	3 × 3	3 × 3	94.33%	93.33%	84.00%	90.55%
10	3 × 3	3 × 3	3 × 3	2 × 2	2 × 2	2 × 2	94.67%	92.33%	91.67%	92.89%
11	3 × 3	3 × 3	2 × 2	3 × 3	2 × 2	3 × 3	91.67%	75.00%	92.00%	86.22%
12	3 × 3	3 × 3	2 × 2	2 × 2	3 × 3	2 × 2	93.33%	93.00%	88.33%	91.55%

**Table 10:** Average CAR of Main Factor-Level Response.

Kernel Size	Main Factor	A	B	C	D	E	F
2 × 2	Average CAR	91.53%	92.76%	91.28%	92.59%	91.59%	92.50%
3 × 3	Average CAR	91.15%	89.93%	91.41%	90.10%	91.09%	90.19%
	Difference	−0.38%	−2.83%	0.13%	−2.49%	−0.50%	−2.31%
	Rank (absolute)	5	1	6	2	4	3

**Table 11:** Image Size Variation after Each Convolution and Max-Pooling Layer.

Original Image Size: 200 × 200	Kernel Size: 2 × 2	Kernel Size: 3 × 3
First layer size after convolution	199 × 199	198 × 198
First layer size after Max-Pooling	99 × 99	66 × 66
Second layer size after convolution	98 × 98	64 × 64
Second layer size after Max-Pooling	49 × 49	21 × 21
Third layer size after convolution	48 × 48	19 × 19
Third layer size after Max-Pooling	24 × 24	6 × 6

**Table 12:** Recommended and Contrast Combinations.

Combination	A	B	C	D	E	F
Recommended combination (A1B1C2D1E1F1)	2 × 2	2 × 2	3 × 3	2 × 2	2 × 2	2 × 2
Contrast combination (A2B2C1D2E2F2)	3 × 3	3 × 3	2 × 2	3 × 3	3 × 3	3 × 3

**Table 13:** CAR of Recommended and Contrast Combinations.

Combination	Test 1	Test 2	Test 3	Test 4	Test 5	Average
Recommended combination (A1B1C2D1E1F1)	96.67%	94.67%	93.00%	96.00%	95.33%	95.13%
Contrast combination (A2B2C1D2E2F2)	91.00%	86.00%	91.33%	84.00%	91.33%	88.73%

## Data Availability

Dataset available on request from the authors.
